# Time trends in atrial fibrillation-related stroke during 2001–2020 in Sweden: a nationwide, observational study

**DOI:** 10.1016/j.lanepe.2023.100596

**Published:** 2023-02-21

**Authors:** Mozhu Ding, Marcus Ebeling, Louise Ziegler, Alexandra Wennberg, Karin Modig

**Affiliations:** a Unit of Epidemiology, Institute of Environmental Medicine, Karolinska Institutet, Stockholm, Sweden; b Max Planck Institute for Demographic Research, Rostock, Germany; c Department of Clinical Sciences, Danderyd Hospital, Karolinska Institutet, Stockholm, Sweden

**Keywords:** Atrial fibrillation, Ischemic stroke, Cohort studies, Oral anticoagulant drugs, Time trends

## Abstract

**Background:**

Great efforts have been made to improve stroke prevention in atrial fibrillation (AF) patients. Meanwhile, incidence of AF is increasing, which may affect the share of AF-related stroke on all strokes. We aimed to examine the temporal trends in the incidence of AF-related ischemic stroke between 2001 and 2020, if it varied by use of novel oral anticoagulant drugs (NOAC), and if the relative risk of ischemic stroke associated with AF changed over time.

**Methods:**

Data from the total Swedish population aged ≥70 years during the period 2001–2020 were used. Annual incidence rate (IR) was calculated for overall and AF-related ischemic stroke which was defined as first-ever ischemic stroke with AF diagnosed up to 5 years before, on the same day, or within 2 months after the stroke event. Cox regression models were performed to examine if the hazard ratio (HR) between AF and stroke changed over time.

**Findings:**

While IR of ischemic strokes declined during 2001–2020, IR of AF-related ischemic stroke remained stable between 2001 and 2010 but showed a consistent decline between 2010 and 2020. The HR of ischemic stroke within 3 years from an AF diagnosis came down from 2.39 (95% confidence interval: 2.31–2.48) to 1.54 (1.48–1.61) over the study period, which was largely explained by a substantial increase in the use of NOAC among AF patients after 2012. Yet, by the end of 2020, 24% of all ischemic strokes had a preceding or concurrent AF diagnosis, which is slightly higher than in 2001.

**Interpretation:**

Even though both the absolute and relative risk of AF-related ischemic stroke declined over the past 20 years, every fourth ischemic stroke in 2020 still had a preceding or concurrent AF diagnosis. This represents a great potential for future gains in stroke prevention among AF patients.

**Funding:**

10.13039/501100004359Swedish Research Council and 10.13039/100010771Loo and Hans Osterman Foundation for Medical Research.

**Research in context**

**Evidence before this study**

We searched Web of Science for articles published until September 1st, 2022, using the terms: “atrial fibrillation”, “ischemic stroke”, “time trends OR temporal trends”, and “oral anticoagulant OR warfarin” with no language or date restrictions. Most studies reported time trends in atrial fibrillation (AF)-related stroke up until 2010 and only one study was based on more recent data, with follow-up until 2016. Although some studies showed a decrease in AF-related ischemic strokes, others have indicated no decline. However, almost all studies only reported number of ischemic stroke admissions that were associated with AF, without estimating how the absolute and relative risk of AF-related stroke have changed over time. Considering temporal changes in age structure due to population aging, current available evidence is not sufficient to draw concrete conclusions as to whether and how the risk of AF-related stroke has changed in the general population, and what impact oral anticoagulant drugs have had on these trends.

**Added value of this study**

To the best of our knowledge, our study is the first to report temporal trends in AF-related ischemic strokes in relation to all ischemic strokes during the past two decades at a national level. Data from high-quality Swedish national registers allowed us to account for population aging and calculate both absolute and relative risk of AF-related stroke using precise population at-risk data, in a way that has not previously been done. We showed that while the incidence of ischemic strokes declined during the past 20 years, the decline in AF-related stroke became apparent only from 2010 onwards, which is partly explained by a great increase in the use of novel oral anticoagulant drugs (NOACs). However, by the end of 2020, one fourth of first-ever ischemic strokes still had a preceding or concurrent AF diagnosis.

**Implications of all the available evidence**

The fact that AF-related ischemic strokes have declined at a pace similar to overall ischemic strokes during the last decade is assuring, especially because the incidence of AF, as well as the awareness and detection of AF, has increased over the same period. Yet, every fourth ischemic stroke still had a preceding or concurrent AF diagnosis, representing a great potential for future gains in stroke prevention among AF patients. NOACs have undoubtedly played a big role in preventing AF-related strokes and contributing to a favorable trend.

## Introduction

Stroke incidence has declined substantially in high-income countries over the past decades.^1^, ^2^, ^3^, ^4^ In Sweden, despite the demographic shift to an aging population, the decline in the incidence of ischemic strokes has been large enough to offset an otherwise increase in the absolute number of stroke cases.^5^ At the same time, incidence and prevalence of atrial fibrillation (AF), a most common risk factor for ischemic stroke, has increased.^6^ AF not only confers 3- to 5-fold increased risk of ischemic stroke, but AF-related strokes are often more disabling and fatal,^7^^,^^8^ resulting in higher rates of institutionalization in long-term facilities. It is not known if the decline in overall ischemic stroke has extended also to AF-related ischemic strokes.

Current evidence on AF-related strokes trends is scarce and inconclusive, with some studies suggesting a stable trend and others indicating a decline.^9^, ^10^, ^11^, ^12^ On the one hand, increased prescription of oral anticoagulants (OAC) in recent years, especially novel oral anticoagulant drugs (NOAC) after 2010, following an AF diagnosis could have led to a decrease in AF-related ischemic strokes. On the other hand, increasing AF incidence has raised concerns of a potential increase in AF-related strokes.^13^ Therefore, investigating temporal trends of AF-related ischemic stroke and how it relates to the use of OAC is of high public health relevance for informing stroke prevention strategies and allocation of healthcare resources.

Sweden is unique in its high-quality registers on population statistics, hospital admissions, and prescribed medications, where nation-wide data have been mandatorily reported and collected over many decades. Utilizing the entire Swedish older population aged 70 years and over, the aim of this study was to 1) examine the temporal trends in the incidence of overall ischemic strokes as well as AF-related ischemic strokes during 2001–2020, 2) investigate how the risk of first-ever ischemic stroke after AF diagnosis changed over calendar time, and 3) the impact of OAC and NOAC use on risk of first-ever ischemic stroke among AF patients over time.

## Methods

### Study population

We derived data from the Swedish Total Population Register^14^ to identify all individuals who were born between 1895 and 1947 and had been living in Sweden since January 1st 1987. Through a unique personal identification number, the Total Population Register is linked to individual-level data from national registers, including the National Patient Register (since 1987), the Cause of Death Register (since 1952), and the Prescribed Drug Register (since 2005). Ethical approval for this study was obtained from the Regional Ethics Committee in Stockholm (2011/136–31/5).

To investigate temporal trends in the incidence of first-ever ischemic stroke, we included all individuals aged ≥70 years without a history of overall stroke on January 1st each calendar year during the period 2001–2020. Because this study focused on first-ever ischemic stroke in relation to AF, we applied a long washout period as the data allowed to ensure individuals were stroke-free for as long as possible at the time of inclusion. Therefore, individuals needed to be living in Sweden for at least 14 years (washout period) for identification of stroke history – e.g., people identified on January 1st, 2001, should have been living in Sweden and stroke-free at least since 1987, etc. All individuals included in each calendar year were followed from January 1st until first-ever stroke, emigration, death, or December 31st.

To explore how the risk of first-ever ischemic stroke following an AF diagnosis have changed over time, both in an absolute sense and in relation to not having AF, four different models were fitted, each referring to one calendar period. In this way the risk of stroke and AF, as well as the covariates were allowed to vary between the time periods. We identified all individuals aged ≥70 years who were diagnosed with new-onset AF from January 1st, 2006 until December 31st, 2017. These AF patients were categorized into four groups according to time of diagnosis: January 1st, 2006 to December 31st, 2008, January 1st, 2009 to December 31st, 2011, January 1st, 2012 to December 31st, 2014, and January 1st, 2015 to December 31st, 2017. Because the Prescribed Drug Register started on July 1st, 2005, from which medication data was derived, the follow-up in the Patient Register for this analysis started in 2006. All individuals with new-onset AF were followed from the day of AF diagnosis for 3 years for incident ischemic stroke, emigration, death, or end of the 3-year follow-up. Those without new-onset AF during each period were followed from January 1st of each period for 3-year incident ischemic stroke, emigration, death, or end of the 3-year follow-up. Individuals with a history of overall stroke at the start of each period were excluded.

### Ascertainment of stroke and atrial fibrillation

Stroke and AF events were identified through the Swedish National Patient Register and the Cause of Death Register. The National Patient Register contains hospital discharge records from inpatient care at national level since 1987 and data on specialized outpatient care since 2001. Information retrieved from this register includes the dates and discharge diagnoses of each hospital visit which were coded according to the International Classification of Diseases (ICD) system. The National Patient Register has fairly high sensitivity and specificity with regards to stroke and AF.^14^^,^^15^ The Cause of Death Register is a complete register of all deaths in Sweden since 1952, and underlying and contributing causes of deaths were recorded following the ICD system. The following ICD codes were used to identify stroke and AF: ICD-9 434 and ICD-10 I63 for ischemic stroke, and ICD-9 427.3 and ICD-10 I48 for AF. Because the most feared complication of OAC among AF patients is intracranial hemorrhage, secondary analyses were performed to examine the temporal trends for hemorrhagic stroke (ICD-10: I61; ICD-9: 431) in patients with concomitant AF.

Through linkage of these national registers, we were able to identify all first-ever ischemic strokes as well as those that had a preceding or concurrent AF diagnosis. AF-related ischemic stroke was defined as people diagnosed with first-ever ischemic stroke and having an AF diagnosis up to 5 years before, on the same day, or within 2 months after the stroke event. AF diagnosis within 2 months after stroke was included to decrease the likelihood of missing AF-related stroke cases due to late AF diagnosis.^16^ The same definition was applied to hemorrhagic stroke with concomitant AF.

### Ascertainment of comorbidities

History of heart failure (HF), coronary heart diseases (CHD), diabetes, hypertension, and vascular diseases (i.e., myocardial infarction, peripheral artery disease, and aortic plague) were identified from the National Patient Register using the following ICD codes: HF (ICD-9: 402, 404, 425, 428; ICD-10: I110, I130, I132, I27, I280, I42, I43, I515, I517, I528), CHD (ICD-9: 410–414; ICD-10: I20-25), diabetes (ICD-9: 250, 251.D; ICD-10: E10, E11, E13, E14), hypertension (ICD-9: 401–405; ICD-10: I10, I13, I15), myocardial infarction (ICD-9: 410; ICD-10: I21), peripheral artery disease (ICD-9: 440.1, 440.2, 440.8, 440.9, 441, 443.9; ICD-10: I70.1, I70.2, I70.8, I70.9, I71, I73.9), aortic plague (ICD-9: 440.0; ICD-10: I70.0), transient ischemic attack (TIA) (ICD-9: 435; ICD-10: G45), liver disease (ICD-9: 571–573; ICD-10: K70, K74, K75), and kidney disease (ICD-9: 581–583, 753, V420; ICD-10: N03–N05, Q61, Z940). To enable fair comparisons of comorbidity history in different time periods, we only allowed for disease identification at most 5 years before the baseline of each period. CHA_2_DS_2_-VASc score at AF diagnosis was calculated as scoring 1 point each for HF, diabetes, hypertension, vascular diseases, age 65–74, and female sex, and 2 points for age ≥75 years.^17^ Ischemic stroke was excluded from the CHA_2_DS_2_-VASc score in this study, because all individuals with a stroke history were excluded at AF diagnosis.

### Assessment of oral anticoagulant drugs

Beginning in July 2005, information on prescribed medication was available in the National Prescribed Drug Register for all surviving individuals. All prescriptions in this register were coded using the Anatomical Therapeutic Chemical (ATC) system, and prescription of OAC was identified using ATC codes B01AA, B01AD, B01AE, B01AF, and B01AX. Prescription of NOAC was identified using ATC codes B01AE and B01AF. Individuals were considered OAC users if they picked up an OAC from the pharmacy at least once within 3 years after AF diagnosis and before stroke. The vast majority of AF patients had at least two OAC pickups during the 3-year follow-up (93.8% for AF diagnosed 2006–2008, 94.0% for 2009–2011, 94.5% for 2012–2014, and 95.4% for 2015–2017).

### Statistical analysis

Annual incidence rate (IR) of ischemic stroke was calculated as the number of first-ever ischemic strokes during each calendar year divided by the number of person-years at risk for ischemic stroke. Annual IR for AF-related ischemic stroke was calculated as number of first-ever ischemic strokes that were associated with AF divided by number of person-years at risk for ischemic stroke. To make the IR of stroke comparable over 2001–2020, direct age-standardization was applied to account for differences in the age structure of populations over time. The age structure of the population in 2010 was used as the reference, and age-standardized IR was calculated by weighting their respective age-specific rates to the age structure of the reference population. The percentage of AF-related ischemic strokes out of all ischemic strokes in each calendar year was also calculated. The same set of analyses were performed for hemorrhagic strokes.

Cox regression models were used to estimate hazard ratios (HR) and 95% confidence interval (CI) for 3-year incident ischemic stroke in association with AF diagnosed in different time periods (i.e., 2006–2008, 2009–2011, 2012–2014, and 2015–2017), as compared to no AF. P for trend was then calculated to examine whether there is a statistically significant temporal trend in these HRs. All models were first adjusted for age and sex, and then additionally adjusted for history of HF, CHD, hypertension, diabetes, vascular diseases, TIA, liver disease, and kidney disease at the baseline of each period. Sensitivity analyses were performed specifying deaths during the follow-up as a competing risk in Cox regression models.

We then ascertained whether increasing use of OAC and NOAC could have played a role in reducing risk of ischemic stroke after AF diagnosis over time. The proportion of individuals who used any OAC or NOAC within 3 years after AF diagnoses was estimated. Using Cox regression models, risk of 3-year incident ischemic stroke among AF patients diagnosed in periods 2009–2011, 2012–2014, and 2015–2017 was compared to that in 2006–2008. All models were first adjusted for history of comorbidities, and then additionally adjusted for use of OAC and NOAC.

All statistical analyses were conducted using Stata 16.1 (StataCorp LLC, College Station, TX 77845, USA).

### Role of the funding source

The funders of the study had no role in study design, data collection, data analysis, data interpretation, or writing of the report.

## Results

### Temporal trends in the incidence of atrial fibrillation-related strokes

**Fig. 1:**
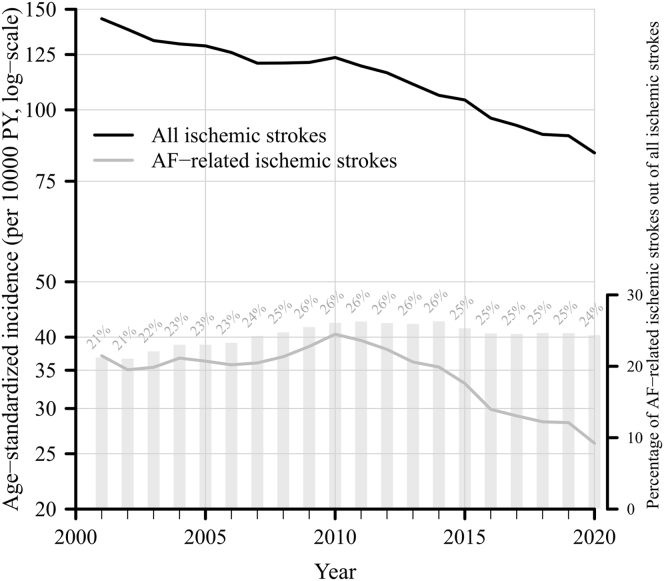
Age-standardized annual incidence rate (per 10,000 person-years) for all ischemic strokes (black line) and atrial fibrillation-related ischemic strokes (gray line), and the percentage of number of atrial fibrillation-related ischemic strokes out of all ischemic strokes (gray bars), between year 2001 and 2020, in the Swedish older population aged ≥70 years. AF = atrial fibrillation; PY = person-years.

Fig. 1 shows the temporal trends in the IR of all ischemic strokes and AF-related ischemic strokes in the entire Swedish population aged ≥70 years between year 2001 and 2020. The IR of ischemic strokes showed a consistent and steep decline over the whole period (age-standardized IR per 10,000 person-years 2001 vs. 2020 = 144 vs. 84). The IR of AF-related stroke remained largely unchanged between 2001 and 2010 (age-standardized IR per 10,000 person-years 2001 vs. 2020 = 37 vs. 40). However, starting from 2010, the IR of AF-related stroke began to decline at a similar pace as that of overall strokes (age-standardized IR per 10,000 person-years 2010 vs 2020 = 40 vs. 26). The proportion of the number of AF-related ischemic strokes out of all ischemic strokes increased from 21% in 2001 to 26% in 2010, after which it remained largely stable until 2020 (Fig. 1). The results were similar between men and women (Fig. S1). Sensitivity analyses excluding ischemic stroke with AF diagnosed within 2 months after stroke showed similar results as the main analyses (Fig. S2). When stratified by 5-year age groups, time trends in the incidence rate of all and AF-related ischemic strokes in each age group was very similar to that in the total population, except for the oldest-old 90–94 and ≥ 95 years where the incidence of both all and AF-related ischemic strokes increased from year 2001–2010 but declined after 2010 (Fig. S3).

The incidence rate of hemorrhagic strokes remained low during the entire study period and showed a slight decline between 2001 and 2020 (age-standardized IR per 10,000 person-years 18 vs. 14) (Fig. S4). The incidence rate of hemorrhagic stroke with concomitant AF was even lower, although it showed an increase between 2001 and 2013 (age-standardized IR per 10,000 person-years 3 vs. 5) and levelled off thereafter.

### Temporal trends in the association between AF and stroke risk

Table 1 presents baseline characteristics of individuals diagnosed with and without new-onset AF in four consecutive time periods. Compared to individuals diagnosed with AF in 2006–2008, those diagnosed in 2015–2017 were more likely to be men, have a history of HF, hypertension, diabetes, kidney disease, and liver disease, and less likely to have a history of CHD and vascular diseases. No individual was classified as 0 (low stroke risk) in the CHA_2_DS_2_-VASc score, and more than 96% were classified as ≥2 (high stroke risk). The mean CHA_2_DS_2_-VASc score at AF diagnosis increased slightly over time, from 3.1 in 2006–2008 to 3.3 in 2015–2017. The age at AF diagnosis declined slightly over time, with median age of 81.1 years (interquartile range 75.1–86.5) in 2015–2017 as compared to 81.6 years (76.3–86.4) in 2006–2008.

**Table 1:** Characteristics of individuals with and without new-onset atrial fibrillation in different time periods.

Characteristics	2006–2008		2009–2011		2012–2014		2015–2017	
	No AF	New-onset AF	No AF	New-onset AF	No AF	New-onset AF	No AF	New-onset AF
No. of subjects	862,871	56,848	860,613	59,385	873,740	62,603	949,488	65,441
Women, n (%)	524,177 (60.8)	30,026 (52.8)	519,189 (60.3)	31,164 (52.5)	521,160 (59.7)	32,426 (51.8)	556,225 (58.6)	33,066 (50.5)
Age at AF (years), median (IQR)	–	81.6 (76.3–86.4)	–	81.7 (76.2–86.8)	–	81.5 (75.7–86.8)	–	81.1 (75.1–86.5)
History of comorbidities								
Heart failure, n (%)	11,773 (1.4)	3948 (6.9)	13,321 (1.6)	4361 (7.3)	14,351 (1.6)	4713 (7.5)	15,465 (1.6)	6839 (10.5)
Coronary heart disease, n (%)	84,900 (9.8)	16,627 (29.3)	87,294 (10.1)	16,705 (28.1)	88,895 (10.2)	16,861 (26.9)	89,510 (9.4)	16,389 (25.0)
Hypertension, n (%)	98,516 (11.4)	22,703 (39.9)	148,697 (17.3)	30,471 (51.3)	200,430 (22.9)	36,207 (57.8)	240,789 (25.4)	39,096 (59.7)
Diabetes, n (%)	56,191 (6.5)	7921 (13.9)	65,490 (7.6)	9192 (15.5)	73,333 (8.4)	10,405 (16.6)	84,032 (8.9)	11,512 (17.6)
Vascular diseases, n (%)	47,615 (5.5)	9691 (17.1)	47,147 (5.5)	9682 (16.3)	47,709 (5.5)	9724 (15.5)	48,777 (5.1)	9589 (14.7)
TIA, n (%)	14,286 (1.7)	2207 (3.9)	14,848 (1.7)	2401 (4.0)	16,861 (1.9)	2689 (4.3)	19,209 (2.0)	2667 (4.1)
Kidney disease, n (%)	3400 (0.4)	590 (1.0)	4185 (0.5)	698 (1.2)	4885 (0.6)	760 (1.2)	5282 (0.6)	937 (1.4)
Liver disease, n (%)	1925 (0.2)	239 (0.4)	2229 (0.3)	281 (0.5)	2728 (0.3)	348 (0.6)	3559 (0.4)	445 (0.7)
CHA_2_DS_2_-VASc score								
Mean (SD)	–	3.1 (1.0)	–	3.2 (1.1)	–	3.2 (1.1)	–	3.3 (1.1)
1, n (%)	–	2179 (3.8)	–	1996 (3.4)	–	2146 (3.4)	–	2404 (3.7)
≥2, n (%)	–	54,669 (96.2)	–	57,389 (96.9)	–	60,457 (96.6)	–	63,037 (96.3)
No. of ischemic stroke during 3-year follow-up	22,933	4025	22,084	3858	20,488	3266	19,923	2524
Age at ischemic stroke (years), median (IQR)	80.7 (76.6–85.7)	84.2 (79.9–88.5)	80.5 (76.3–85.8)	84.4 (79.4–89.5)	80.1 (76.1–85.6)	84.4 (79.1–89.5)	79.4 (75.7–84.9)	83.9 (78.4–89.2)

AF = atrial fibrillation, IQR = interquartile range, SD = standard deviation, TIA = transient ischemic attack.

Temporal trends in the association between AF and 3-year incident ischemic stroke are shown in Table 2. The IR of 3-year incident ischemic stroke among AF patients decreased from 29.9 (95% CI 29.0–30.9) among individuals diagnosed with AF during 2006–2008 to 15.7 (95% CI 15.1–16.3) in those diagnosed during 2015–2017. Compared to no AF, the HR for 3-year incident ischemic stroke was 2.39 (95% CI 2.31–2.48) in individuals diagnosed with AF during 2006–2008, 2.22 (2.14–2.30) during 2009–2011, 1.92 (1.85–2.00) during 2012–2014, and 1.54 (1.48–1.61) during 2015–2017, adjusting for age, sex, HF, CHD, hypertension, diabetes, vascular diseases, TIA, liver disease, and kidney disease at the baseline of each time period. The decline in these HRs was statistically significant (p for trend <0.0001). Accounting for competing risk of death in Cox regression models resulted in slighted attenuated estimates (Table S1).

**Table 2:** Incidence rate and hazard ratio (95% confidence interval) for 3-year incident ischemic stroke associated with atrial fibrillation diagnosed in different time periods.

Time periods	No. of 3-year ischemic stroke	IR for 3-year ischemic stroke per 1000 PY (95% CI)	HR (95% CI)	
			Model 1	Model 2
No AF during 2006–2008	22,933	9.4 (9.3–9.6)	1.00 (Ref.)	1.00 (Ref.)
AF diagnosed 2006–2008	4025	29.9 (29.0–30.9)	2.72 (2.63–2.81)	2.39 (2.31–2.48)
No AF during 2009–2011	22,084	9.1 (8.9–9.2)	1.00 (Ref.)	1.00 (Ref.)
AF diagnosed 2009–2011	3858	27.4 (26.5–28.3)	2.54 (2.45–2.63)	2.22 (2.13–2.30)
No AF during 2012–2014	20,488	8.3 (8.2–8.4)	1.00 (Ref.)	1.00 (Ref.)
AF diagnosed 2012–2014	3266	21.7 (21.0–22.4)	2.18 (2.10–2.26)	1.92 (1.85–2.00)
No AF during 2015–2017	19,923	7.4 (7.3–7.5)	1.00 (Ref.)	1.00 (Ref.)
AF diagnosed 2015–2017	2524	15.7 (15.1–16.3)	1.77 (1.70–1.85)	1.54 (1.48–1.61)
*p for trend*			*<0.0001*	*<0.0001*

Model 1: adjusted for age and sex. Model 2: adjusted for age, sex, and history of heart failure, coronary heart disease, hypertension, diabetes, vascular diseases, transient ischemic attack, liver disease, and kidney disease. AF = atrial fibrillation, IR = incidence rate, HR = hazard ratio, CI = confidence interval, PY = person year.

### Temporal trends in the use of anticoagulant drugs and its impact on the association between AF and stroke

The use of any type of OAC among AF patients increased only slightly from 83.7% in 2006–2008 to 87.2% in 2015–2017 (Table 3). However, a greater increase was seen in the use of NOAC – from 0.1% in 2006–2008 to 64.0% in 2015–2017. Without adjustment of OAC and NOAC, individuals with new onset AF in 2009–2011, 2012–2014, and 2015–2017 were respectively 10%, 29%, and 47% less likely to develop incident ischemic stroke over the 3-year follow-up, compared to those with new onset AF in 2006–2008 (HR = 0.90, 95% CI: 0.86–0.94; HR = 0.71, 95% CI: 0.68–0.74; HR = 0.53, 95% CI: 0.50–0.55, respectively) (Table 3). However, further adjusting for use of NOAC attenuated the HR substantially for 2012–2014 and 2015–2017 (HR = 0.81, 95% CI: 0.77–0.84; HR = 0.82, 95% CI: 0.77–0.87) and there was no longer any difference in the risk of ischemic stroke between these two time periods.

**Table 3:** Use of anticoagulant drugs and hazard ratio (95% confidence interval) for 3-year incident ischemic stroke among atrial fibrillation patients diagnosed in 2009–2011, 2012–2014, and 2015–2017, as compared to 2006–2008.

AF diagnosed in different time periods	Use of any OAC within 3 years after AF diagnosis, n (%)	Use of NOAC within 3 years after AF diagnosis, n (%)	HR (95% CI) for 3-year ischemic stroke		
			Model 1	Model 2 (Model 1+ OAC)	Model 3 (Model 1 + NOAC)
2006–2008 (n = 56,848)	47,552 (83.7)	34 (0.1)	1.00 (Ref.)	1.00 (Ref.)	1.00 (Ref.)
2009–2011 (n = 59,385)	50,594 (85.2)	1053 (1.8)	0.90 (0.86–0.94)	0.91 (0.87–0.95)	0.91 (0.87–0.95)
2012–2014 (n = 62,603)	53,391 (85.3)	13,230 (21.1)	0.71 (0.68–0.74)	0.72 (0.69–0.76)	0.81 (0.77–0.84)
2015–2017 (n = 65,441)	57,047 (87.2)	41,859 (64.0)	0.53 (0.50–0.55)	0.55 (0.52–0.57)	0.82 (0.77–0.87)

Model 1: adjusted for age, sex, and history of heart failure, coronary heart disease, hypertension, diabetes, vascular diseases, transient ischemic attack, liver disease, and kidney disease. Model 2: Model 1 + use of any oral anticoagulant drugs within 3 years after AF diagnosis. Model 3: Model 1 + use of novel anticoagulant drugs within 3 years after AF diagnosis. HR = hazard ratio; CI = confidence interval; OAC = oral anticoagulant drugs; NOAC = novel oral anticoagulant drugs; AF = atrial fibrillation.

Similar results and patterns were also found among age groups, although NOAC were less commonly used in AF patients aged ≥80 years than in those aged 70–79 years (57.7% vs. 70.8% in 2015–2017) (Table S1). In age group 70–79 years, adjusting for NOAC largely explained the risk reduction of stroke in AF diagnosed in 2015–2017 compared to those diagnosed in 2006–2008, while in age group ≥80 years, this risk reduction was only partially explained by use of NOAC.

## Discussion

In this large population-based study using health records from the entire Swedish population aged ≥70 years, we found that the incidence of AF-related ischemic stroke declined by 35% between 2001 and 2020. The decline is particularly prominent after 2010, around the same time when NOACs were introduced to the Swedish market. This observation was further confirmed in the analyses showing that the use of NOACs increased substantially among AF patients during the 2010s, and that this largely explained the decrease in AF-related stroke risk from 2012 and onwards. Yet, despite all these improvements, our data show that by the end of 2020 around one-fourth of all first-ever ischemic stroke events still had a preceding or concurrent AF diagnosis.

Data reporting temporal trends in AF-related strokes are mixed and mostly based on data from more than ten years ago. A decrease in the incidence of cardioembolic stroke associated with AF between 1985 and 2006 was reported in France.^10^ In the Framingham Heart Study, a 74% decline in the risk of stroke following AF onset was observed between 1958-2007.^18^ Another US study using the Medicare database also found a 50% decline in ischemic stroke rates among AF patients from 1992 to 2007, in parallel with a doubling use of warfarin.^19^ It is worth noting that OAC prescription for stroke prevention among AF patients was implemented during the 1990s. Therefore, the fast decline in stroke risk following AF diagnosis around the 1990s in previous studies could reflect improvements from an era in which OAC was not used and paroxysmal AF was not considered a relevant diagnosis. On the contrary, a US study using healthcare databases in the Olmsted County showed no change in the risk of ischemic stroke and use of OAC following AF diagnosis between 2000 and 2010.^11^ Similarly, one UK study reported no decline in the incidence of AF-related ischemic stroke from 2002 to 2012.^12^ Data after 2010 is scarce; only one recent study aggregating several UK national databases supported a decrease in the weekly hospital admission rates of AF-related stroke between 2011 and 2016.^9^

Discrepancies in previous findings could be due to differences in clinical practice among regions and countries, as well as study time periods. In addition, almost all studies only reported number of stroke admissions that were associated with AF, without estimating how the absolute and relative risk of AF-related stroke have changed over time.

To our knowledge, our study is the first to simultaneously report time trends in the IR of all ischemic strokes and AF-related ischemic strokes in the total older population, allowing direct comparisons. In line with other studies,^1^, ^2^, ^3^, ^4^ we found a steep and consistent decline in the incidence of ischemic strokes after 2000. On the other hand, the IR of AF-related ischemic stroke did not start to decline until 2010, suggesting that stroke prevention in AF patients before 2010 (in the era of warfarin) was not enough to make the overall ischemic stroke incidence decline. In our survival analyses, AF was to a lesser extent associated with incident stroke in 2012–2014 and 2015–2017 as compared to 2006–2008, in parallel with a dramatic increase in the use of NOACs. These survival analyses also indicate that the uptake of NOACs was at least partially the reason behind the marked decrease in AF-related stroke from 2010 and onwards, complementary to the descriptive trend figure. Since 2011, NOACs such as dabigatran, rivaroxaban, and apixaban have been available in Sweden as alternatives to warfarin. Notably, the European Society of Cardiology (ESC) guideline on AF management was published in August 2012, and recommended NOACs as preferable to Vitamin K antagonist (e.g., warfarin) for ischemic stroke prevention in AF patients.^20^ One study using Stockholm Healthcare Analysis Database reported a 12.5% increase in the prescription of NOACs 5 months after the ESC guideline was published,^21^ in line with our results. The encouraging trend in AF-related ischemic stroke risk and use of NOACs between 2010 and 2020 in our study reflect a continued and long-lasting effort of disseminating evidence-based medicine from clinical trials to everyday practice in Sweden. In addition, national reimbursement decisions on NOACs, as well as overall better cost-effectiveness of NOACs compared to warfarin,^22^ may also have aided these favorable trends.

It is worth noting that the increasing use of NOACs was not the only explanation to the decrease in stroke risk among AF patients, as the risk of stroke remains 18% smaller in 2015–2017 compared to 2006–2009 after adjusting for NOAC use. Factors such as better adherence to warfarin and NOAC, improved lifestyle factors, better rate and rhythm control in AF patients, and risk factor management in AF including pharmaceutical management of co-existing cardiometabolic disorders may have played a role, as well. Moreover, attenuated HRs after NOACs adjustment can also reflect unmeasured confounding related to both NOACs prescriptions and risk of stroke. For instance, reduced alcohol consumption and smoking cessation could have resulted in less contraindication to use of NOACs among more recently diagnosed AF patients and thus a lower risk of stroke.

Large-scale clinical trials have demonstrated apparent superiority of NOACs (e.g., dabigatran and apixaban) over warfarin in terms of better stroke prevention and less intracranial bleeding, as well as advantage of a wider therapeutic window and lack of a need for regular monitoring.^23^, ^24^, ^25^ Yet, it has been argued that clinical trials mainly include younger individuals with fewer comorbidities and major bleeding associated with NOACs. However, recent observational cohort studies of older AF patients revealed that NOACs were associated with similar risk of ischemic stroke as warfarin but lower risk of major bleeding,^26^, ^27^, ^28^ indicating that NOACs are safe in older adults with multi-morbidities. Our data showed that the IR of hemorrhagic strokes with concomitant AF increased until 2013 and remained stable thereafter. The increase before 2013 could reflect intracranial bleeding associated with warfarin when it was still the dominating drug, while the flattening after 2013 may reflect an effect of NOAC implementation, even though NOACs may have been introduced at different paces in Swedish regions. Nevertheless, it should be noted that the absolute risk of hemorrhagic strokes remained very low during the entire study period, i.e., <6 cases per 10,000 PY. The use of NOACs were higher in people aged 70–79 than in those aged ≥80 years. However, 58% of AF patients aged ≥80 years were using NOACs and 84% were using at least one type of OAC in 2015–2017. Notably, their risk of ischemic stroke after AF onset showed a comparable decline over time to that of the younger age group. For age group 90–94 and ≥ 90 years, there was however an increase in the IR of AF-related stroke between 2000 and 2010. Although the reason behind this increase is not entirely clear, one explanation could be that during 2000s a large proportion of oldest-old AF patients were not treated with OAC but rather with antiplatelet drugs,^29^ possibly resulting in an increase in AF-related stroke incidence.

Despite the encouraging patterns of a declining incidence in AF-related ischemic stroke, our results showed that by the end of 2020, one out of four ischemic strokes in people aged ≥70 years still had a preceding or concurrent AF diagnosis. This shows that there is still room for improvement in risk reduction of stroke in AF patients. In addition to increasing NOAC coverage, improved detection and diagnostics of AF in at-risk populations may be justified.^30^

The strength of this study lies in its size and nation-wide information on prescribed medications and hospital admissions of AF and stroke subtypes, as well as detailed and precise data on population at risk. We are also the first to report up-to-date data until the end of 2020. However, some limitations should be considered. First, data underlying this study is part of a larger project concerning aging and health where available data is from ages 70 years and over. Yet, as the risk of both AF and stroke increase with age, most cases of AF and ischemic stroke in Sweden occur after age 70 (around 75%).^29^^,^^31^ Our findings should therefore be primarily generalized to older populations. For early-onset AF and stroke, e.g., those occurred during midlife or earlier, it remains to be seen whether the same pattern can be observed. Second, because of the observational nature of this study and the fact that AF-related stroke is defined based on the temporality of diagnostic codes, we cannot draw any causal inference that ischemic strokes with a preceding or concurrent AF diagnosis are directly a consequence of AF. For example, comorbidities other than those adjusted for in the survival analyses could have accounted for a share of incident stroke following an AF diagnosis. Moreover, due to lack of brain and vascular imaging data, we could not determine the underlying etiologies of the ischemic strokes and hence a proportion of strokes with a proceeding AF diagnosis might have etiologies other than cardiac embolism. However, the proportion of cardiac embolism as the underlying ischemic stroke etiology in relation to other major etiology subtypes is increasing with population aging,^32^^,^^33^ therefore lowering the risk of this misclassification. Third, the awareness and detection of AF in the general population has improved over the study period, as well as the availability and intensity of electrocardiogram investigations in stroke patients without previously diagnosed AF. It is thus likely that AF-related strokes are more often underestimated in the early stage than in the later stage of the study period. Moreover, as the identification of both stroke and AF was based on diagnostic codes, the IRs in this study are likely somewhat lower than those from studies using clinical examinations. Furthermore, primary care data was not available in this study. Previous data from Stockholm region has shown that around 12% of AF patients were only diagnosed in primary care in 2006–2010.^34^ Another Swedish study showed that the prevalence of previously known AF among ischemic stroke patients increased from 29% when hospital discharge was used to 38% when all healthcare contacts were considered.^35^ Since we follow individuals longitudinally, some of the individuals with AF diagnosed in primary care might be included at a later stage when they visit specialized care or hospital. However, it is still likely that some cases of AF diagnosed in primary care only are missed in our study. Waiting times in long-term electrocardiogram investigation among stroke patients could also have led to some missed AF diagnoses. Taking these limitations together, it is likely that the IR of AF-related stroke and share out of all strokes were underestimated throughout the period. Lastly, because we defined use of medication as picking up the prescribed drug at least once within 3 years after AF diagnosis, the use of OAC and NOAC could have been slightly overestimated among AF patients in each time period. Nevertheless, the numbers reported in our study are comparable to previous reports examining use of NOAC using Swedish data.^36^

Taken together, although ischemic stroke incidence has declined in the last 20 years in Sweden, the decline in the incidence of AF-related ischemic stroke became apparent only from 2010 onwards and is to a large extent explained by the increasing use of NOACs. However, by the end of 2020, one out of four first-ever ischemic strokes still had a preceding or concurrent AF diagnosis, representing a great potential to further prevent stroke in older adults with AF.

## Contributors

MD and KM conceptualized and designed the study. MD and ME conducted the data analyses, and MD drafted the manuscript. All authors contributed to the interpretation of results and critically reviewed the manuscript. MD and ME have directly accessed and verified the underlying data reported in the manuscript.

## Data sharing statement

Due to the General Data Protection Regulation in Sweden, the pseudo-anonymized personal data underlying this study cannot be shared publicly. Access to the data and the codes for data analyses can be permitted to external researchers after ethical vetting and establishment of a collaboration agreement. Contact the corresponding author for questions about data sharing (MD). Aggregated data underlying Fig. 1 is available at https://osf.io/5h7cy/?view_only=c1f30f20f7d546dc942fbbb6c707ee41.

## Declaration of interests

We declare no competing interests.

## Appendix A Supplementary data

Supplementary materials

STROBE-checklist
