# Supplementary material for: Time trends in atrial fibrillation-related stroke during 2001–2020 in Sweden: a nationwide, observational study
Source: Lancet Reg Health Eur. 2023 Feb 21;28:100596. doi: 10.1016/j.lanepe.2023.100596 (PMC10173271; doi:10.1016/j.lanepe.2023.100596)
Supplement: Supplementary materials [file mmc1.pdf]

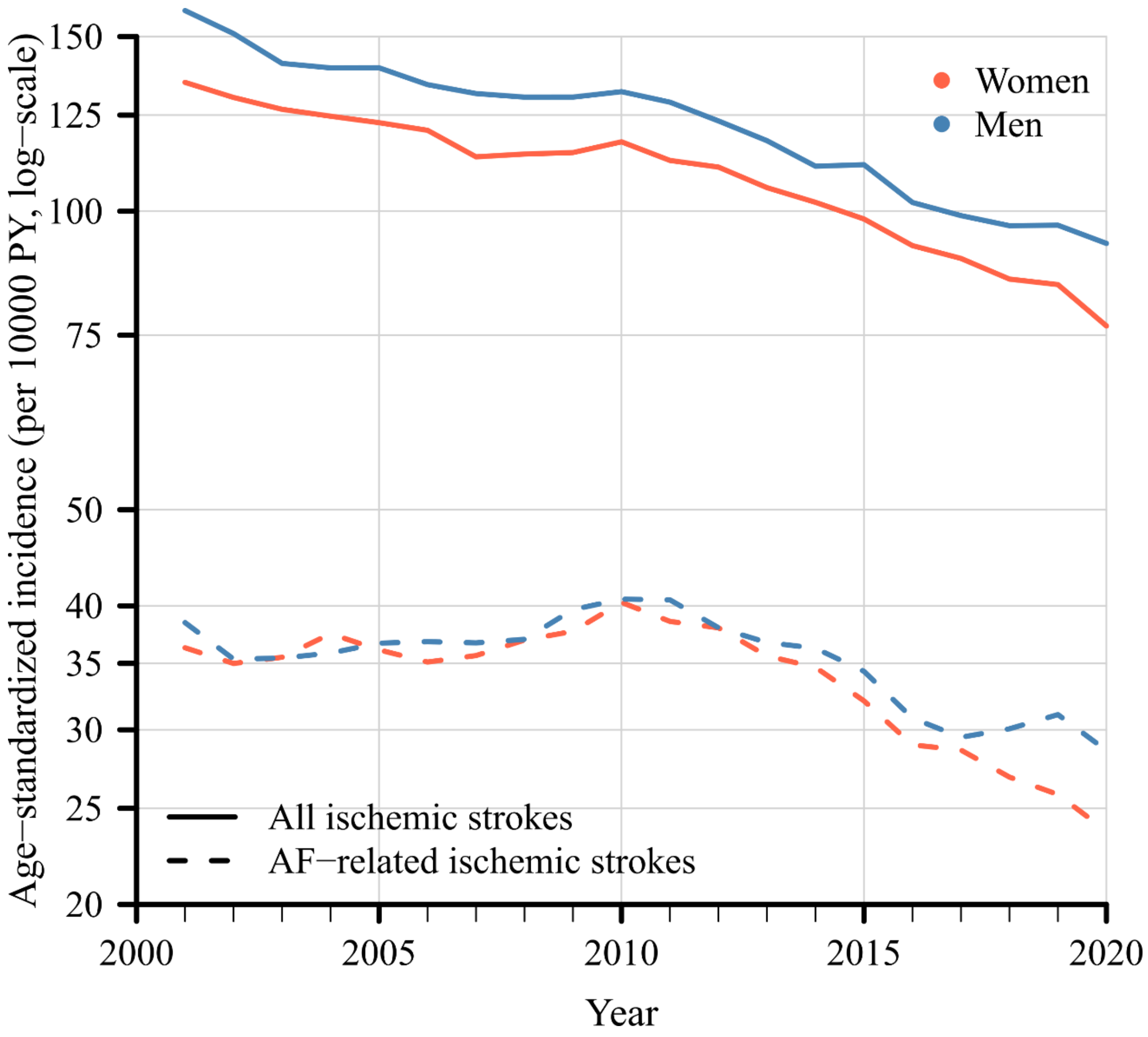

Figure S1. Age-standardized annual incidence rate (per 10000 person-years) for all ischemic strokes (solid line) and atrial fibrillation-related strokes (dashed line) stratified by sex, between year 2001 and 2020, in the Swedish older population aged ≥70 years. PY=person-years, AF=atrial fibrillation.

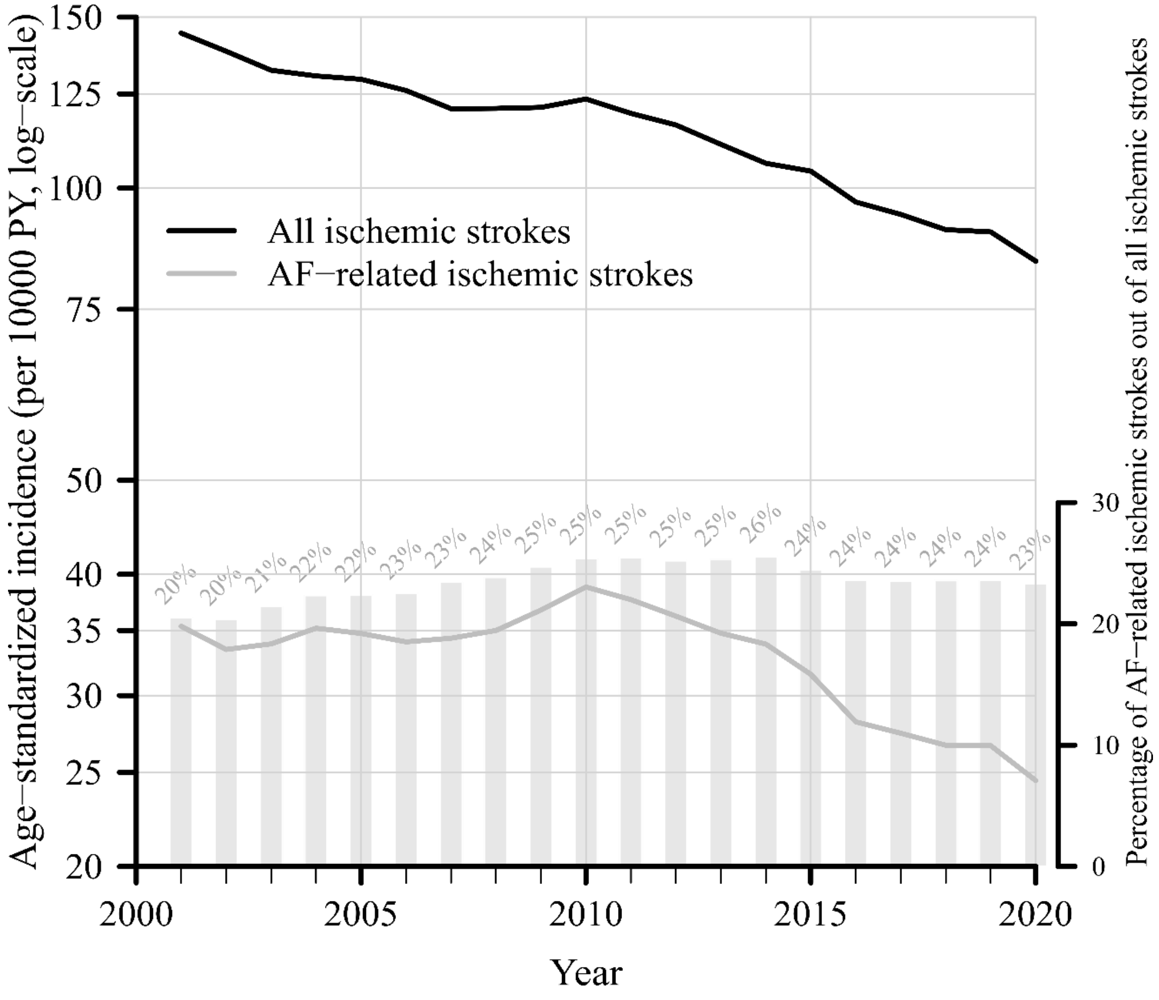

Figure S2. Age-standardized annual incidence rate (per 10000 person-years) for all ischemic strokes (solid line) and atrial fibrillation-related strokes (dashed line) stratified by sex, between year 2001 and 2020, in the Swedish older population aged ≥70 years. Atrial fibrillation-related strokes were defined as first-ever ischemic strokes with AF diagnosed within 5 years before or on the same day as the stroke event. PY=person-years, AF=atrial fibrillation.

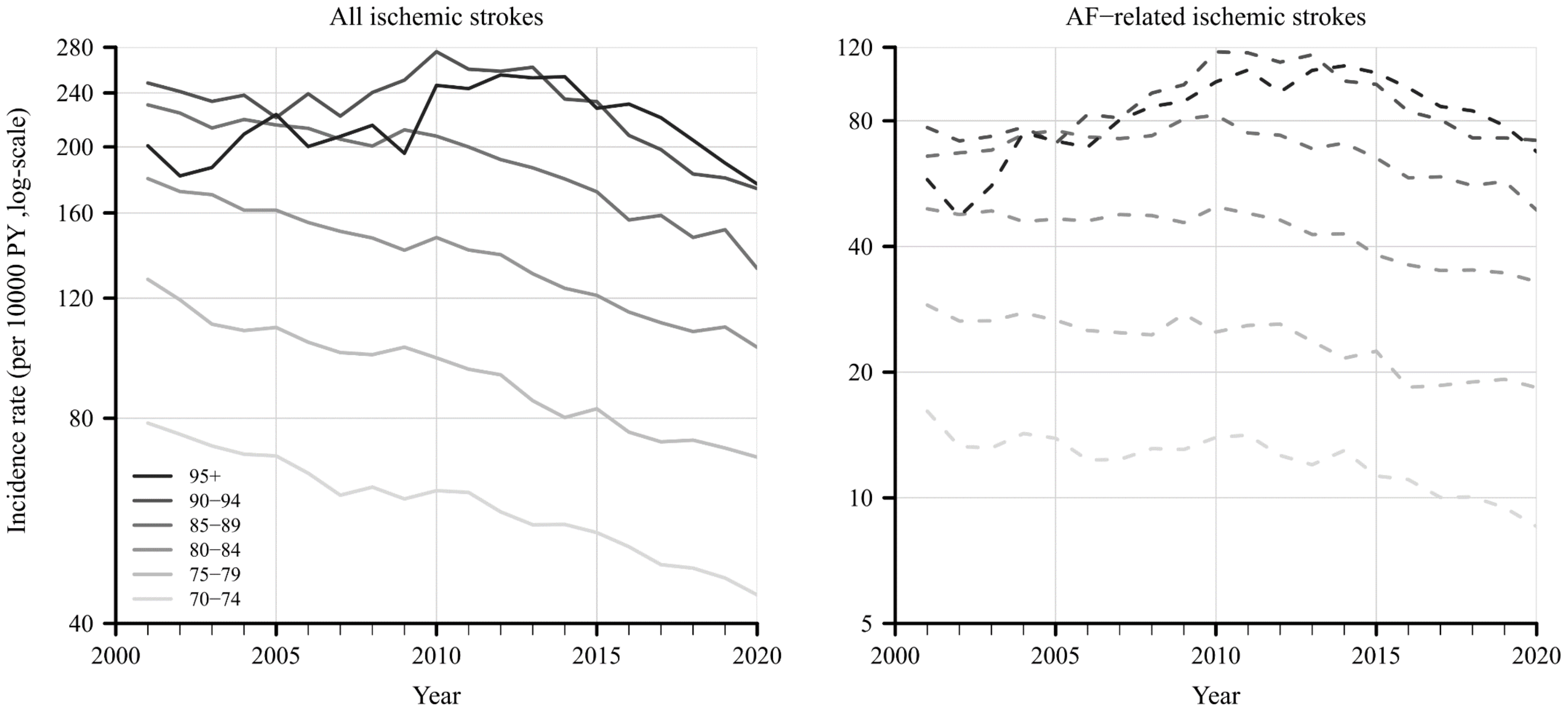

Figure S3. Age-standardized annual incidence rate (per 10000 person-years) for ischemic strokes (solid line, left) and atrial fibrillation-related ischemic strokes (dashed line, right) stratified by age groups, between year 2001 and 2020, in the Swedish older population aged ≥70 years. PY=person-years, AF=atrial fibrillation.

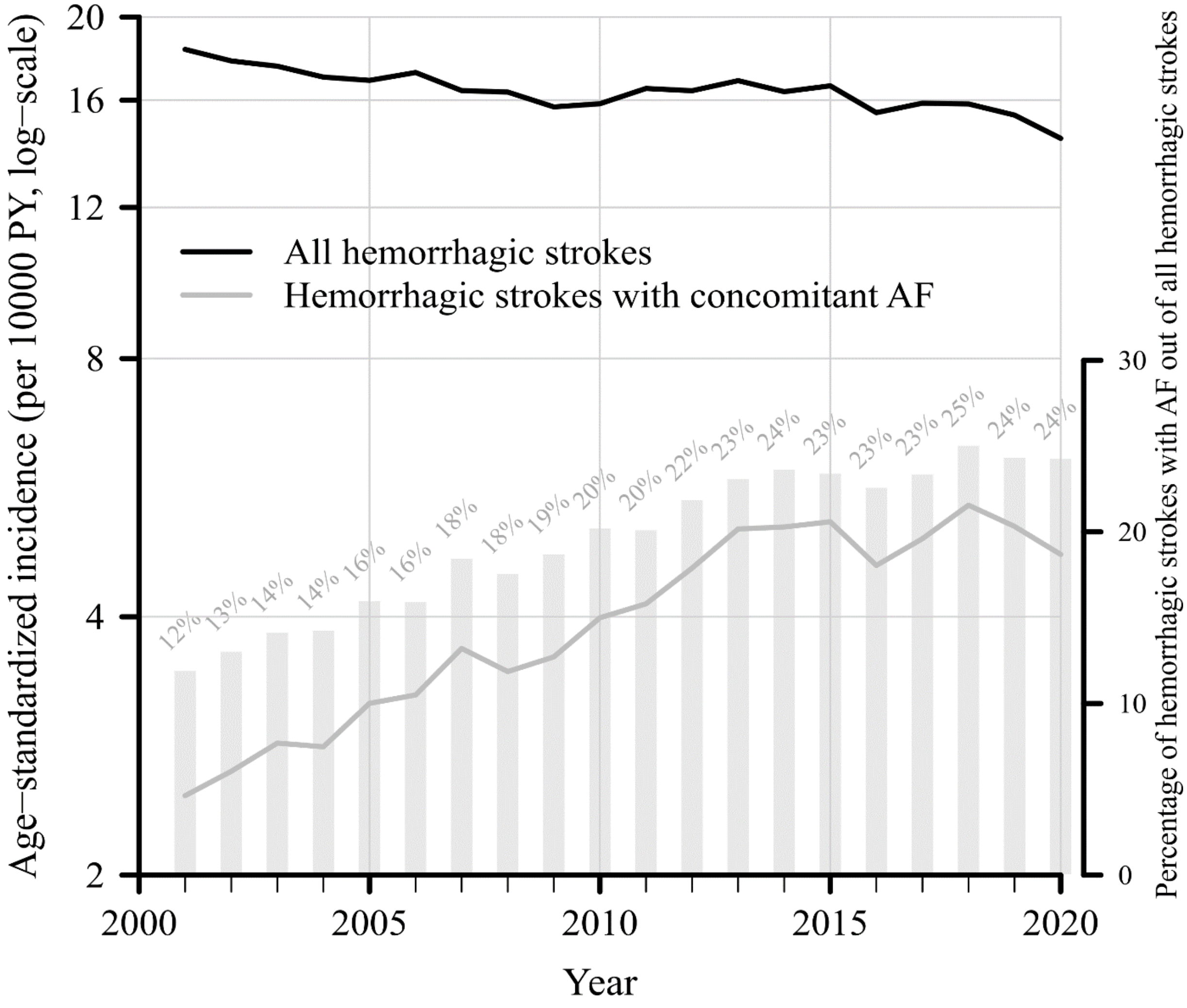

Figure S4. Age-standardized annual incidence rate (per 10000 person-years) for all hemorrhagic strokes (black line) and hemorrhagic strokes with concomitant atrial fibrillation (grey line), and the percentage of number of hemorrhagic strokes with concomitant atrial fibrillation out of all hemorrhagic strokes (gray bars), between year 2001 and 2020, in the Swedish older population aged ≥70 years. PY=person-years, AF=atrial fibrillation.

Table S1. Subdistribution hazard ratio (95% confidence interval) for 3-year incident ischemic stroke associated with atrial fibrillation diagnosed in different time periods.

**Time periods**	**No. of subjects**	**No. of 3-year ischemic stroke**	**No. of deaths as competing events**	**Subdistribution hazard ratio (95% confidence interval)**
No AF during 2006-2008	862871	22933	118250	1.00 (Ref.)
AF diagnosed 2006-2008	56848	4025	18843	2.13 (2.05-2.21)
No AF during 2009-2011	860613	22084	111323	1.00 (Ref.)
AF diagnosed 2009-2011	59385	3858	19689	2.01 (1.90-2.13)
No AF during 2012-2014	873740	20488	108603	1.00 (Ref.)
AF diagnosed 2012-2014	62603	3266	20397	1.75 (1.65-1.85)
No AF during 2015-2017	949488	19923	110111	1.00 (Ref.)
AF diagnosed 2015-2017	65441	2524	20499	1.35 (1.27-1.43)
p for trend				*<0.0001*

The models were adjusted for age, sex, and history of heart failure, coronary heart disease, hypertension, diabetes, vascular diseases, transient ischemic attack, liver disease, and kidney disease. The subdistribution hazard ratios were derived from Cox regression models specifying deaths during the follow-up as competing risk. AF=atrial fibrillation.

Table S2. Use of anticoagulant drugs and hazard ratio (95% confidence interval) for 3-year incident ischemic stroke among atrial fibrillation patients diagnosed in 2009-2011, 2012-2014, and 2015-2017, as compared to 2006-2008, stratified by age groups.

			**HR (95% CI) for 3-year incident ischemic stroke**		
**AF diagnosed in different time periods**	**Use of any OAC within 3 years after AF diagnosis, n (%)**	**Use of NOAC within 3 years after AF diagnosis, n (%)**	Model 1	Model 2 (Model 1+ OAC)	Model 3 (Model 1 + NOAC)
**Age group 70-79**					
2006-2008 (n=24010)	21401 (88.1)	26 (0.1)	1.00 (Ref.)	1.00 (Ref.)	1.00 (Ref.)
2009-2011 (n=25177)	22721 (89.6)	712 (2.8)	0.95 (0.88-1.03)	0.98 (0.90-1.06)	0.96 (0.89-1.04)
2012-2014 (n=27698)	25070 (90.0)	7388 (26.5)	0.72 (0.67-0.79)	0.75 (0.69-0.81)	0.85 (0.78-0.92)
2015-2017 (n=31183)	28785 (91.7)	22224 (70.8)	0.56 (0.52-0.61)	0.60 (0.55-0.65)	0.93 (0.84-1.03)
**Age group ≥80 years**					
2006-2008 (n=31949)	26151 (80.3)	8 (0.0)	1.00 (Ref.)	1.00 (Ref.)	1.00 (Ref.)
2009-2011 (n=33639)	27873 (81.9)	341 (1.0)	0.87 (0.83-0.92)	0.88 (0.83-0.91)	0.88 (0.83-0.93)
2012-2014 (n=34470)	28321 (81.5)	5842 (16.8)	0.70 (0.67-0.75)	0.72 (0.68-0.76)	0.79 (0.74-0.83)
2015-2017 (n=33700)	28262 (83.0)	19635 (57.7)	0.51 (0.48-0.54)	0.52 (0.49-0.56)	0.77 (0.71-0.82)

Model 1: adjusted for age, sex, and history of history of heart failure, coronary heart disease, hypertension, diabetes, vascular diseases, transient ischemic attack, liver disease, and kidney disease. Model 2: Model 1 + use of any oral anticoagulant drugs within 3 years after AF diagnosis.

Model 3: Model 1 + use of novel anticoagulant drugs within 3 years after AF diagnosis. HR=hazard ratio; CI=confidence interval; OAC=oral anticoagulant drugs; NOAC=novel oral anticoagulant drugs; AF=atrial fibrillation
